# Starch Nanocomposite Films: Migration Studies of Nanoparticles to Food Simulants and Bio-Disintegration in Soil

**DOI:** 10.3390/polym14091636

**Published:** 2022-04-19

**Authors:** Florencia Ortega, Pablo Sobral, Jorge L. Jios, Valeria B. Arce, María Alejandra García

**Affiliations:** 1 CIDCA (Centro de Investigación y Desarrollo en Criotecnología de Alimentos), Facultad de Ciencias Exactas, Universidad Nacional de La Plata (UNLP)-CONICET La Plata, 47 y 116 S/N°, La Plata 1900, Argentina; fortega@biol.unlp.edu.ar; 2 Departamento de Química, Facultad de Ciencias Exactas, Universidad Nacional de La Plata (UNLP), 47 y 115, La Plata 1900, Argentina; pablosobral@biol.unlp.edu.ar (P.S.); jljios@quimica.unlp.edu.ar (J.L.J.); varce@quimica.unlp.edu.ar (V.B.A.); 3 Laboratorio UPL (UNLP-CIC), Campus Tecnológico Comisión de Investigaciones Científicas de la Provincia de Buenos Aires, Cno. Centenario entre 505 y 508, Manuel B. Gonnet 1897, Argentina; 4 CIOp (Centro de Investigaciones Ópticas), (UNLP)-CICPBA Universidad Nacional de La Plata, Camino Centenario e/505 y 508, Gonnet 1897, Argentina

**Keywords:** silver nanoparticles, starch nanocomposite films, release kinetic, food simulants, bio-disintegration

## Abstract

In this work, films containing AgNPs were obtained by different green synthesis techniques (AgNP in situ and AgNP L). The inclusion of nanoparticles in the starch matrix improved both mechanical and barrier properties. The migration of AgNPs from the nanocomposite material to three food simulants (water, 3% *v*/*v* acetic acid and 15% *v*/*v* ethanol) was studied. The experimental data were fitted by using different widely accepted mathematical models (Fickian, Ritger and Peppas, and Weibull), indicating that the AgNP migration followed a complex mechanism. The silver concentration (mg Ag per kg of simulant) that was released from the nanocomposite films was higher for the samples with AgNPs in situ than for those containing AgNP L. Likewise, the maximum release value (0.141 mg/dm^2^ for AgNPs in situ in acetic acid simulant) was lower than the limits proposed by the legislation (European Commission and MERCOSUR; 10 and 8 mg/dm^2^, respectively). The replacement of conventional plastic materials by biodegradable ones requires the evaluation of bio-disintegration tests in soil. In this sense, a period of 90 days was necessary to obtain ≥50% weight loss in both nanocomposite films. Additionally, the bio-disintegration of the samples did not contribute with phytotoxic compounds to the soil, allowing the germination of fast-growing seeds.

## 1. Introduction

Due to the environmental impact of plastic materials derived from non-renewable sources that are used to store, protect and preserve foods, more effort is being made to develop packaging from more environmentally friendly materials. These new materials based on renewable sources, such as polysaccharides, proteins, and natural active agents of plant or marine origin, present biodegradable or compostable properties in an attempt to reduce the environmental impact associated with petroleum-based plastics [1,2]. With this purpose, starch is one of the most commonly studied, readily available carbohydrates, obtainable from renewable sources at a relatively low cost. Starch films present very good oxygen barrier capacity, but due to their hydrophilic nature, films exhibit water sensitivity and poor water vapor barrier properties. Combining the films with nanotechnology would improve these characteristics by providing adequate water barrier capacity as well as mechanical properties [3,4,5,6].

The development and characterization of nanocomposite films includes different types of nanoparticles. There are numerous investigations that describe how they are obtained by conventional and green synthesis, in both synthetic and biodegradable matrices [7,8,9,10,11], although those related to the study of migration processes are scarce [12,13]. Moreover, the legislation for the use of these materials is still under review, and this is necessary in order to advance in this field, due to the growing use of nanocomposite materials in various areas. This development was paralleled by public safety concerns about nanomaterials in general and it is crucial in the case of nanomaterials used in polymers for food packaging applications [13]. Will the nanoparticles be able to move within the nanocomposite materials towards the food contact surface and finally be released into the food? This question will have to be resolved in the first instance. This process could occur by a migration via Fickian diffusion, as with conventional additives or other mechanisms such as degradation of the polymer matrix by mechanical abrasion, hydrolysis or swelling of polymer chains, among others [13,14]. To analyze and quantify the transfer of nanoparticles to food products and simplify their subsequent detection, food simulants are used instead of real food. The food simulants vary in terms of their chemical properties, thus representing several different food types: hydrophilic (water based), lipophilic (fatty foods) or amphiphilic (foods with watery and fatty properties) [15].

On the other hand, the biodegradability of a material is a measure of its ability to be degraded by environmental conditions, particularly the enzymatic activity of microorganisms, such as fungi and bacteria. Generally, biodegradable materials are decomposed into CO_2_ and H_2_O under aerobic conditions and methane gas, CO_2_, H_2_S and H_2_, among others, under anaerobic conditions [16,17]. However, not all biodegradable polymers are compostable, since compostability implies biodegradation by biological processes at a rate consistent with other known compostable materials, leaving visually indistinguishable and non-toxic residues, according to Tampau et al. [2]. Therefore, the evaluation of compostability includes three phases: disintegration, biodegradation and ecotoxicity.

In previous studies [18,19], silver nanoparticles obtained by in situ green synthesis (AgNP in situ) were coupled to starch films, to obtain nanocomposite materials with improved mechanical and barrier properties at a concentration of 143 ppm. In addition, these films showed good antimicrobial activity against the microorganisms responsible for foodborne diseases. On the other hand, in Ortega et al. [20], nanocomposite starch films containing AgNPs synthesized by the active compounds presented in fresh lemon juice (AgNP L) were developed. The concentration that improved the mechanical, barrier and antimicrobial properties for these films was 71.5 ppm AgNP L. Therefore, considering these results and their further application as food packaging, nanocomposite films with 143 ppm AgNP in situ and 71.5 ppm AgNP L were selected. The aims of the present work were to evaluate the possible silver migration from the previously selected nanocomposite materials in comparison to three food simulants (water, 3% *v*/*v* acetic acid and 15% *v*/*v* ethanol) and to study their bio-disintegration under composting conditions.

## 2. Materials and Methods

### 2.1. Materials

Silver nitrate (AgNO_3_) and maltose (reducing agent) were purchased from Biopack (Biopack, Buenos Aires, Argentina). Lemons (*Citrus limon*) used were grown in La Plata City, Argentina (34°56′26″ S 57°59′44″ W). Native corn starch with 25% amylose was provided by Unilever (Unilever, Buenos Aires, Argentina). The average molecular weight was 2 × 10^4^ gmol^−1^ for amylose and 2 × 10^5^–1 × 10^6^ gmol^−1^ for amylopectin [21]. Glycerol (Anedra, Buenos Aires, Argentina) was used as a plasticizer. The rest of the reagents used were of analytical grade.

### 2.2. Nanocomposite Starch Films

Nanocomposite starch films were obtained by the casting method. Materials containing silver nanoparticles in situ (AgNP in situ) were synthesized according to the methodology optimized by Ortega et al. [18,19]. Filmogenic suspensions with 3% *w*/*v* native corn starch were gelatinized at 90 °C for 20 min, then 10 mL of AgNO_3_ (6.5 × 10^−4^ M) and 20 mL of maltose (1.3 × 10^−3^ M) solutions were added, and these were kept under stirring for 20 min at 90 °C. Films including silver nanoparticles obtained by active compounds of lemon juice (AgNP L) were prepared as specified by Ortega et al. [20]. The volume of 1920 μL of AgNP L synthesized at 90 °C by Ortega et al. [20] was mixed with 100 mL of 3% *w*/*v* corn starch suspensions. Then, nanocomposite suspensions were gelatinized at 90 °C for 20 min.

In both cases, after gelatinization, the nanocomposite filmogenic suspensions were cooled at 50 °C and 30% *w*/*w* glycerol was added as plasticizer and then dried in an air-forced convection oven (50 °C for 5 h).

The final concentration of nanoparticles in the active materials was expressed in mg of Ag per kg dried film and corresponds to 143 and 71.5 ppm for AgNPs and AgNP L, respectively.

The characterization of the films (thickness, water vapor permeability and mechanical properties) was carried out according to Ortega et al. [18,19,20].

Infrared spectroscopy by Fourier transform with attenuated total reflectance (ATR-FTIR) was used to identify the functional groups present in the samples, as well as their interactions. ATR-FTIR spectra of the films were acquired with a Nicolet-iS10 FTIR spectrometer (Thermo Scientific, Waltham, MA, USA) with attenuated total reflection. Spectra were achieved from 32 accumulated scans at 4 cm^−1^ resolution in the range: 4000–400 cm^−1^. At least five replicates of each sample were analyzed using the Omnic 9 software (Thermo Scientific, Waltham, MA, USA).

### 2.3. Migration Test

The possible AgNP migration was evaluated by the total immersion of the nanocomposite films (AgNPs 143 ppm and AgNP L 71.5 ppm) in three different food simulant media (water, 15% *v*/*v* ethanol and 3% *v*/*v* acetic acid), selected according to the Res. Conj. N° 140 and N° 526, 17.9.2001, Res. GMC MERCOSUR N° 32/97 and N° 33/97. To maintain the area–volume ratio proposed by Kim et al. [22], films with 1.2 × 2.7 cm^2^ were cut and put into tubes containing 13 mL of the corresponding food simulant. The assays were carried out in duplicate at 35 °C [23] in an orbital shaker (Orbit-Environ Shaker, Lab-Line Instruments, USA) at 125 rpm and then, samples were taken at 1, 2, 4, 8, and 24 h, and 3, 5 and 7 days of contact. After the mentioned contact times, the films were removed and the simulants were analyzed by an inductively coupled plasma–optical emission spectroscopy system (ICP–OES).

#### Quantification of Silver Content by ICP–OES

The quantification of silver content was performed according to Yu et al. [24] with some modifications. After the migration assay and before the ICP–OES determination, a microwave-assisted mineralization of the sample was performed to destroy organic matter and for homogenization purposes. The test solutions were treated with HNO_3_ (65% *w*/*w*) and H_2_O_2_ (100 vol). The alcoholic simulant samples were conditioned for 24 h at 37 °C to allow the evaporation of the organic component of the medium. In the case of acidic simulant, to reduce the organic matter, the sample was placed with the digestion reagents inside hermetically sealed vessels and a pre-digestion was carried out at a low temperature in an oven for 24 h. Finally, the microwave-assisted digestion was carried out by raising the temperature at 180 °C and leaving it overnight until the vessels cooled; then, they were filtered and led to a volume of 25 mL. The determinations were performed with an ICP–OES model ICPE-9820 (Shimadzu, Kyoto, Japan) coupled with an ASX-520 autosampler (CETAC Technologies, Omaha, NE, USA), while the digestions were performed with a QLABPro Questron Microwave Digestor System (Questron Technologies Corp., Ontario, Canada). The experimental data were analyzed using the InfoStat 2020 software (InfoStat Group, FCA, National University of Cordoba, Córdoba, Argentine) [25]. In all cases, the goodness of fit to the proposed model and the parameters corresponding to each model were estimated.

### 2.4. Mathematical Approach

The migration term is used to refer to the transfer of components (additives or contaminants in some cases) from materials to foods (or simulants) with which they are in contact, under normal conditions of use, processing and storage, and/or in the equivalent test conditions [15].

Diffusion is one of the most studied processes. When analyzing the migration of NPs from a polymeric matrix, other mechanisms can be found, such as surface desorption, ion dissolution, and swelling, among others. The diffusion of a substance in a polymer generally obeys Fick’s second law [26] (Equation (1)):

(1) $$\frac{\partial C_{A}^{P}}{\partial t}=D_{A}^{P}\frac{\partial^{2}C_{A}^{P}}{\partial x^{2}}$$

where *C_A_* represents the local migrant concentration of the additive *A*, *t* is the time, *x* is the lineal direction of the mass transport and *D_A_* is the diffusion coefficient of *A* substance. Considering the boundary conditions and the geometry of the material (a flat plate of thickness *δ*), the solution can be expressed as follows (Equation (2)):

(2) $$\frac{M_{t}}{M_{\infty }}=1-\sum_{x=0}^{\infty }\frac{8}{{\left(2n+1\right)}^{2\;}\pi^{2}}e\left[-\frac{{\left(2n+1\right)}^{2}}{\delta^{2}}Dt\right]$$

where *M_t_* (ppm) is the total amount of the substance that diffuses at time *t* (s), *M_∞_* (ppm) is the amount corresponding at infinite time, *δ* is the film thickness (cm) and D the diffusion coefficient (cm^2^/s). In this equation an apparent diffusion coefficient (Dapp) was proposed, since the films with hydrophilic characteristics, such as starch films, swell in contact with aqueous solvents [27]. In the present work, this condition is assumed, although no nomenclature change has been made.

Simplifying the expression for short times and considering the solution of Equation (2) for a semi-infinite medium:

(3) $$\frac{M_{t}}{M_{\infty }}=4\sqrt{\frac{Dt}{\pi \delta^{2}}}$$

This expression (Equation (3)) indicates that Fickian diffusion in a thin film is characterized by an initial dependence of compound transport that varies with *t*^1/2^. The short time approximation is valid until the release of 60% of the total component capable of diffusing.

Ritger and Peppas [28], developed a semi-empirical model which relates the release of the active compound to the contact time through an exponential equation and allows the differentiation of the release mechanism by diffusion from an anomalous or non-Fickian behavior

(4) $$\frac{M_{t}}{M_{\infty }}=k\;t^{n}$$

where *k* is a kinetic constant associated with the rate at which the process occurs, and *n* is the diffusional exponent indicative of the transport mechanism. This model is considered valid as long as the active compound released is less than 60%, that is, *Mt/M∞* must be < 0.6. It is well known as the Power’s law model.

Another alternative to describe the release mechanisms is through the Weibull equation, which is an empirical model

(5) $$\frac{M_{t}}{M_{\infty }}=1-exp\left(-at^{b}\right)$$

with *a* and *b* constants. The parameter *a* defines the time scale of the process while *b* parameter defines the shape of the curve: *b* = 1 corresponds to an exponential function; *b* > 1 defines a sigmoid curve with an inflection point; and *b* < 1 describes a function that resembles a parabola in short times followed by an exponential for the rest of the curve [29].

### 2.5. Evaluation of the Films’ Bio-Disintegration

The bio-disintegration of films in soil were studied in composting conditions, using fertile soil as substrate according to the methodology described in ASTM D5988-03 [30]. Film samples (3 × 3 cm^2^) were placed in plastic meshes and buried at 5 cm depth from the surface to ensure aerobic degradation. Previously, the films’ moisture content was determined according to Ortega et al. [18,19]. The containers (120 cm^3^) were stored under controlled temperature and moisture conditions (20 °C and 60% RH) and daily irrigated with distilled water to maintain soil moisture. The bio-disintegration was evaluated through weight loss (%) monitoring of the samples throughout the degradation period. In all cases, extracted samples were visually inspected and photographed. Also, at the end of the assay the rest of disintegrated films were examined by scanning electron microscopy (SEM) using a FEI QUANTA 200 SEM (FEI Company, Hillsboro, OR, USA) with an Apolo 40 electron detector and acceleration voltage of 10 kV.

#### Ecotoxicity Estimation by Seeds Growth

To evaluate if the disintegration products of nanocomposite films affect the compost quality, lettuce seeds (*Lactuca sativa*) growth was evaluated conforming to Gautan and Kaur [31]. At the end of the assay, the seeds were planted in different pots (120 cm^3^) containing the compost remains of the disintegrated samples, after homogenizing them. The pots were watered daily and the plants were allowed to develop outdoors (La Plata city, Argentina, 34°56′26″ S 57°59′44″ W). The ability of the seeds to germinate and develop seedlings was determined. Unused compost was used as a control, the tests were performed at least in triplicate.

### 2.6. Statistical Analysis

All assays were performed at least in duplicates, with individually prepared and casted films as replicated experimental units as described in each determination. Systat software (SYSTAT, Inc., Evanston, IL, USA) version 13.0 and InfoStat version 2020 [25] were used for multifactor analysis of variance as well as linear and non-linear regressions. Differences in properties of films and formulations were determined by Fisher’s Least Significant Difference (LSD) test using a significance level of α = 0.05.

## 3. Results and Discussion

### 3.1. Nanocomposite Starch Films Characterization

Previously to obtain the nanocomposite films, silver nanoparticles were characterized by spectroscopy UV-vis, TEM and Z potential. The AgNPs synthesized in situ into the filmogenic suspension presented an average size of 14.2 ± 4.4 nm, while that obtained by using active compounds of lemon juice was 5.5 ± 0.8 nm, and both showed high stability. Also, the addition of AgNPs did not affect the filmogenic capacity of the corn starch suspension [18,19,20].

As it is well known, both water vapor permeability (WVP) and mechanical properties depend on film thickness among other factors. Film thickness was significantly affected (*p* < 0.05) by AgNPs incorporation (Table 1). This result was in agreement with those reported by Kanmani and Rhim [32].

On the other hand, it was observed that, in both cases, the AgNPs improved the WVP and the mechanical properties compared to the control starch films (Table 1). The addition of the nanoparticles (AgNP in situ and AgNP L) significantly (*p* < 0.05) decreased the WVP with respect to the control due to an increase in the matrix tortuosity, which reduces the diffusion of water molecules through the film [19,20].

Regarding mechanical properties, a significant (*p* < 0.05) increase for nanocomposite materials with respect to the control films was observed (Table 1), which is in agreement with other authors’ findings [33,34]. The in situ synthesis of AgNPs into starch films reinforced the matrices, which was evidenced by the higher tensile strength (TS, MPa) and elastic modulus (EM, MPa) of nanocomposite films. These results were also observed when adding silver nanoparticles obtained by active compounds of lemon juice into the starch matrices [20].

In previous works, the antimicrobial capacity of nanocomposite films containing AgNP in situ [18,19] and AgNP L [20] against different microorganisms responsible for foodborne diseases was demonstrated. The nanocomposite films containing 143 ppm AgNPs in situ showed contact inhibition on strains of *E. coli*, and *Salmonella*, and *S. aureus*, while for films with AgNP L, it was observed that Gram-negative bacteria were more sensitive than Gram-positive. Similar results were informed by Zhang and Jiang [34] and Cano et al. [35].

### 3.2. Study of the Migration of Silver Nanoparticles from Nanocomposite Starch Films

One of the most important steps during the development of new materials for food packaging is the study of the possible migration of compounds from the container towards the food product. The migration, in this case of AgNPs, can be strongly influenced by the interaction between these and the polymeric matrix, as well as by the possible interactions of the migrant with the food (or food simulant) with which it is in contact. Usually, it is suggested that the migration of compounds may be conducted by a diffusion process, swelling and relaxation to the materials’ polymeric chains [27,36,37,38].

**Figure 1:**
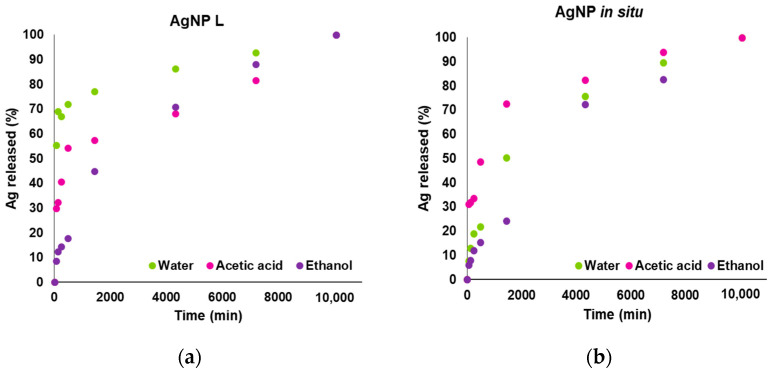
Normalized silver release profiles for the films incorporating AgNP L 71.5 ppm (**a**) and with 143 ppm AgNPs synthesized in situ (**b**), for the three food simulants tested: water, 3% *v*/*v* acetic acid and 15% *v*/*v* ethanol.

Figure 1 shows the silver release profiles from nanocomposite films in contact with the food simulants tested (water, 3% acetic acid and 15% ethanol). Experimental data were normalized with respect to the equilibrium concentration of Ag in each simulant (M∞). Films containing AgNP L (Figure 1a) presented an increased release rate at short times (0 to 8 h) in comparison with those including AgNPs synthesized in situ. Although the migration test is carried out under the conditions standardized by the legislation (MERCOSUR, Resolution MERCOSUR N° 36/92 and Res. Conj. 140 and 526/01 [39]), these do not represent the real situation occurring in a foodstuff. In fact, they overestimate the migration of nanoparticles, because when films are used as packaging, only one side of the material is in contact with the packaged product. In this sense, Vilela Dias et al. [40] stressed that when the diffusion occurs only from one material side, the release ratio is four times lower than when it takes place from both sides. In Figure 1b, the corresponding curves to the AgNP in situ release show a double sigmoid shape at short times, suggesting that the release mechanisms would not agree with a clearly Fickian process. Some authors suggest that the second sigmoid of the release curves corresponds to the existence of slow relaxation phenomena of the polymeric matrix, induced by swelling. These relaxation processes are related to the finite times that the macromolecular chains need to respond to the osmotic swelling pressure and to rearrange themselves by accommodating the solvent molecules that penetrate into the polymeric matrix [38,41].

#### Analysis of Silver Nanoparticles Release Kinetics

In order to estimate the kinetics of silver release from the nanocomposite starch films, the release data were fitted to the mathematical models previously mentioned. The kinetic fitting results are displayed in Table 2. For the first model, the diffusion coefficient (D) was estimated by adjusting the experimental data with Equation (2 (D_1_) and Equation (3 (D_2_) for short contact times (Mt/M∞ < 0.6). The obtained values were similar for the samples containing AgNP L in an acidic and alcoholic medium. In addition, for films with AgNPs, in situ D_1_ and D_2_ values presented the same magnitude order in aqueous and acidic medium. According to Vilela Dias et al. [40], this result indicates that 60% of the diffusion occurs at short contact times between the film and the simulant and Equation (3) can be used to describe this process. However, the correlation coefficient (R^2^) for short contact times was low, so it does not represent the goodness of fit, mainly for the acidic simulant where the R^2^ values were 0.65 and 0.52 for AgNP L and AgNP in situ samples, respectively. For AgNP L samples, although R^2^ presents an acceptable value for short contact times in a hydrophilic medium, the D was obtained by adjusting only a few points, thus the estimated value would not be statistically valid.

The presence of other mechanisms in the migration process (Figure 1) could be responsible for the difference between the experimental data and the fitting curves, because the D are obtained from mathematical expressions assuming only a diffusive behavior and in fact, the release mechanism is more complex and depends on the characteristics of the matrix under study. Therefore, the derived information from the diffusion coefficient values is only indicative. On the other hand, some authors suggest that the dissolution of metal particles into ions cannot be dismissed, as it is becoming an important release mechanism in certain cases [42].

As was previously mentioned, the movement of active compounds from polymeric materials can occur by different processes, which are not taken into account in Fick’s second law. The Ritger and Peppas model analyzes these more complex behaviors and, depending on the values that *n* takes, different transport or release mechanisms can be defined [28]. Table 2 shows the diffusional exponent (*n*) and the kinetic constant (*k*) values, as well as the mean square error (MSE) for analyzed systems. For the films containing AgNP L, in both aqueous and acidic medium, *n* ≤ 0.5 suggesting that the released mechanism of silver nanoparticles was mediated by a Fickian diffusion, while in 15% *v*/*v* ethanolic medium, *n*= 0.61 indicates that the release of the active compound is produced by an “anomalous diffusion” mechanism, since it is in the range of 0.5 < *n* < 1. In this situation, the diffusion process is a combination of Fickian and non-Fickian diffusion, where the phenomenon of relaxation of the polymeric chains that accompanies the diffusion process can occur. Also, for the samples with AgNP in situ in aqueous medium a similar result was observed, since *n* value was 0.59, while the nanoparticles released in acidic and alcoholic medium were by a clearly diffusive mechanism (*n* was 0.23 and 0.43, respectively).

Analyzing the *k* parameter in aqueous medium (Table 2), the obtained value was higher for samples with AgNP L, indicating that the silver release was faster than for AgNP in situ materials at short times. In acidic and 15% ethanolic medium, non differences were observed. Regarding MSE-obtained values, the release profiles for the nanocomposite films with AgNP L in water and ethanol could be satisfactorily adjusted with the Ritger and Peppas model.

Although the calculations indicate that the Ag release mechanism is diffusive for the samples containing AgNP in situ in acetic and ethanolic media (fitted to short times the experimental data by the Power law model), when observing Figure 1b it can be seen that the curve presents a double sigmoidal section at short times. Thus, cannot rule out the presence of a complex migration mechanism that both involves swelling and relaxation of the polymeric chains.

The Weibull model has two parameters, *a* (defines the time scale of the process) and *b* (refers to the shape of the curve). According to this model, the value of *b* also makes reference to the release mechanism. Papadopoulou et al. [36] reported that *b* values between 0.35 and 0.75 correspond to release by diffusion, while if they are in the range of 0.75 to 1, the release mechanism will be a combination of swelling and relaxation of the polymer chains. Finally, if *b* > 1, it is possible that a complex mechanism governs the release of the active component from the material to the simulant medium. When this model was applied to study the mechanism of Ag release, the sample containing AgNP L in the aqueous medium could not be adjusted because the *b* parameter was lower than 0.35 (*b* = 0.22, Table 2). However, with respect to the 3% *v*/*v* acetic acid medium, the model predicted a diffusive release mechanism that agrees with what was previously found by the Ritger and Peppas model. For the 15% *v*/*v* ethanol simulant, the fit by the Weibull model showed a swelling and relaxation of the polymer chains mechanism, which can be compared to that observed by applying Equation (3) for short contact times.

When studying the films with AgNP in situ in aqueous medium, the *b* value was 0.74 (Table 2) indicating a diffusive release mechanism, but we cannot reject that some contribution from the swelling and relaxation of the chains, since this behavior appears for *b* values between 0.75 and 1. Additionally, the Ritger and Peppas model predicted an anomalous mechanism. In acidic medium *b* = 0.74 is in agreement with the previous model (Ritger and Peppas) and the Ag migration would occur by diffusion. Finally, the samples immersed in 15% ethanol presented a complex release mechanism (*b* > 1); although it does not coincide with that proposed by previous models, the double sigmoid shape present in the release profile should be taken into account (Figure 1b).

**Figure 2:**
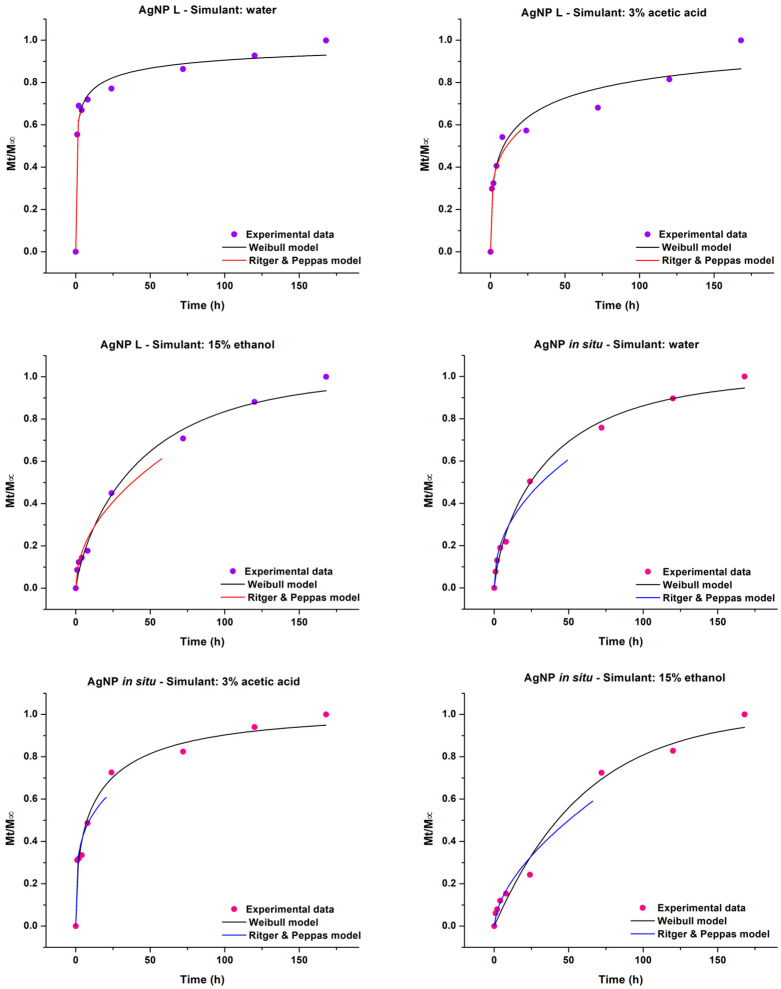
Normalized silver migration profiles for the films containing AgNP L and AgNP in situ with the fit obtained by the Ritger and Peppas and Weibull models for the different simulants tested (water, 3% *v*/*v* acetic acid and 15% *v*/*v* ethanol).

Figure 2 shows the normalized release data together with the fitting curve of the Ritger and Peppas and Weibull model for the three simulants assayed.

The knowledge of the release process of active compounds from the film matrix to food is a determining factor in the development of antimicrobial packaging and most of these studies are carried out using simulation systems, without taking into account the potential effects of these in real foods [40]. In these conditions, the applied models indicated that the films containing AgNP L release Ag to the medium more easily. 

**Figure 3:**
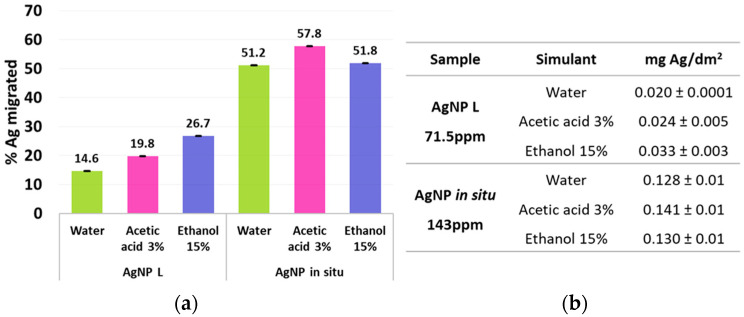
Ag migration percentage (**a**) and Ag content released (**b**) after 7 days of sample immersion in the simulants tested: water, 3% *v*/*v* acetic acid and 15% *v*/*v* ethanol.

In relation to this, the percentage of Ag migration, with respect to the initial content, after 7 days of sample immersion in the simulating medium, was calculated considering the mg of Ag in the material that was used for the test (film of 3.24 cm^2^) and the mg of Ag that migrated to the volume of the simulant (13 mL) at 7 days. As is shown in Figure 3, this content was much lower for the films with AgNP L. Nevertheless, if analyzed according to the nanoparticles’ diameter, although AgNPs in situ would be expected to migrate less due to their larger size (14.2 ± 4.4 nm), the AgNP L (5.5 ± 0.8 nm) probably present a better capping with the different constituents of lemon juice compared to those in situ, where only maltose (reducing agent) and starch (capping and polymer constituent of the matrix) participate in the synthesis. In addition, this behavior may be associated with the characteristics of film matrices. Ortega et al. [20] reported that the presence of citric acid provided by the lemon juice may act as a plasticizer and cross-linking agent, thus slowing down the dissolution process of the matrix, and therefore, the migration and/or dissolution of the AgNPs.

**Figure 4:**
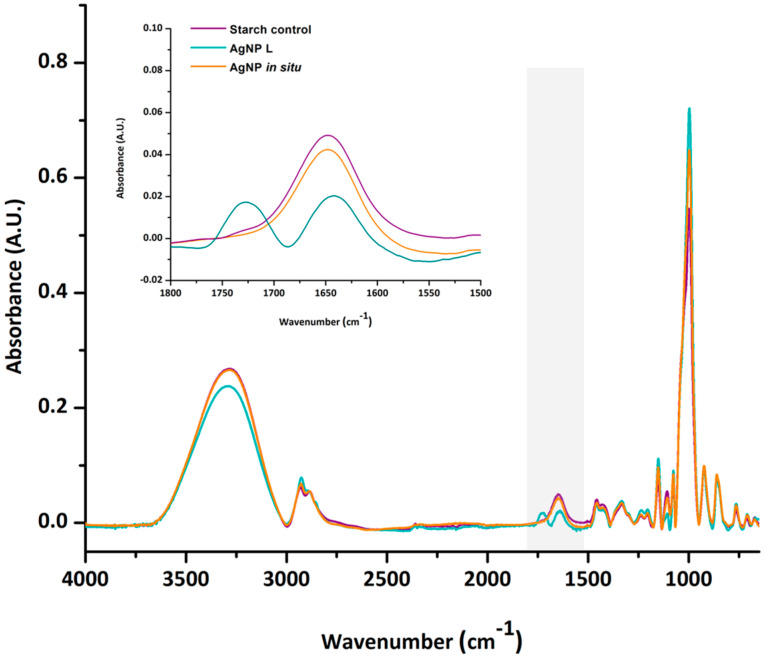
ATR-FTIR spectra of the control and nanocomposite films containing AgNP in situ and AgNP L.

This hypothesis was confirmed through ATR-FTIR spectroscopic analysis. Figure 4 shows the ATR-FTIR spectra of the samples; no great differences were observed in terms of the absorption bands present in the control and nanocomposite films (AgNP in situ and AgNP L), except for that located at 1729 cm^−1^, which is only present in the films with AgNP L. This peak corresponds to the vibrations of the C-O bonds of the carboxyl and ester carbonyl groups, confirming the formation of the ester bond due to the crosslinking of the starch matrix by citric acid. Similar results were informed by Reddy and Yang [44] and Shi et al. [45]. Likewise, the incorporation of AgNP L leads to a decrease in the band at 3290 cm^−1^ attributed to O-H vibration (Figure 4), indicating that these groups are involved in the formation of the ester bond.

The overall migration limit has been defined to ensure that materials do not transfer high quantities of substances which, even if they are safe, could bring about an unacceptable change in the composition of the food [46]. This has established the overall migration limits for food contact materials as 10 mg/dm^2^ or 60 mg/kg of simulant, while the Argentinian regulation [39] set the maximum migration as 8 mg/dm^2^. Regarding this, after 7 days of the immersion of the films into the selected food simulants, it was found that for all cases, the maximum value was lower than the standard established (Figure 3b).

According to Pocas and Franz [47], the information available on the toxicokinetic and toxicological profiles of nanomaterials is limited, and the characterization, detection and measurement of nanoparticles in food and food matrices to assess exposure are still under development. In addition, the possibility of the transfer of nanoparticles to food with processes other than migration should be considered, since the conditions of use may introduce new factors that deviate from the conditions assumed in the mathematical models used.

### 3.3. Evaluation of the Bio-Disintegration of Films in Soil

The disintegrability in composting conditions represents an interesting and attractive property for packaging applications that simulate the post-use of plastics, since it allows evaluating their degradation in natural environments [48,49]. According to Gautam and Kaur [31], the biodegradable materials are those capable of being degraded in natural environments, mainly by enzymatic activity of the microorganism (fungi and bacteria) in the presence (aerobic degradation) or absence (anaerobic degradation) of oxygen giving carbon dioxide, water, inorganic compounds, methane and biomass. To achieve polymer biodegradation, microorganisms first need to cleave the polymer chains in order to reduce their molecular weight and make their transport to cells, where most biochemical processes take place. To break down polymeric materials, microorganisms excrete extracellular enzymes. Then, aerobic or anaerobic deterioration occurs if the material is biodegradable [12].

To determine the physical changes in the samples’ films subjected to the disintegration test, they were periodically removed and examined both visually and by optical microscopy.

**Figure 5:**
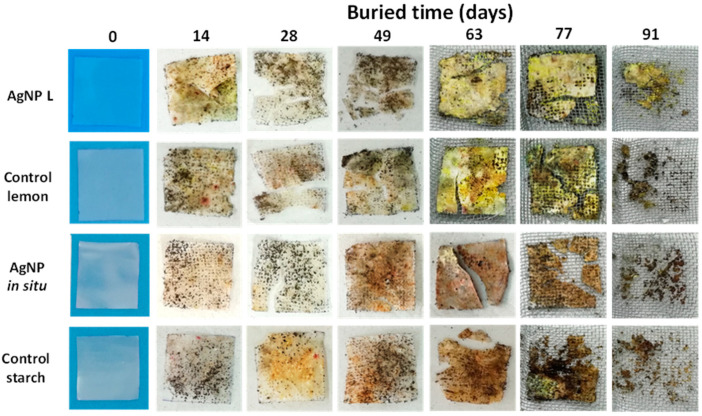
Micrographs of the bio-disintegration process of control and nanocomposite films with AgNP L and AgNP in situ.

The visual appearance of the control samples (control starch and control lemon), and the nanocomposite starch films obtained with AgNP in situ and with the incorporation of 71.5 ppm of AgNP L, after remaining buried for a certain time is shown in Figure 5. As mentioned previously, all the films were initially flexible, colorless and transparent [18,19,20]. Soil contains many types of microorganisms that bring about physical changes which are important indicators of biodegradation. In addition, the visual changes observed (film opacity increase, Figure 4) are also an indicator of microbial action facilitated by the film moisture uptake from the surrounding soil [50,51]. After incubation in soil at 20 °C for 14 days, cracks were observed in both films with AgNP L and the corresponding control. In addition, it was possible to perceive color and opacity changes, which suggests that the process of the disintegration of the materials by the fungi and microorganisms present in the soil began [51]. Likewise, after 28 days of storage, the bio-disintegration of the control starch films and those with AgNP in situ was visible, since water absorption of the films facilitates the microbial attack, causing cracks in the materials. In addition, it was observed that the disintegration of the samples containing extracts of lemon juice required less time compared to the other samples, because the organic compounds present in the lemon would be a richer source of nutrients for microorganisms of the soil and therefore facilitate the degradation of the material. Similar results were reported by Luchese et al. [23] when working with cassava starch films containing orange residues, and by Medina-Jaramillo et al. [52], who added yerba mate extracts to cassava starch films.

**Figure 6:**
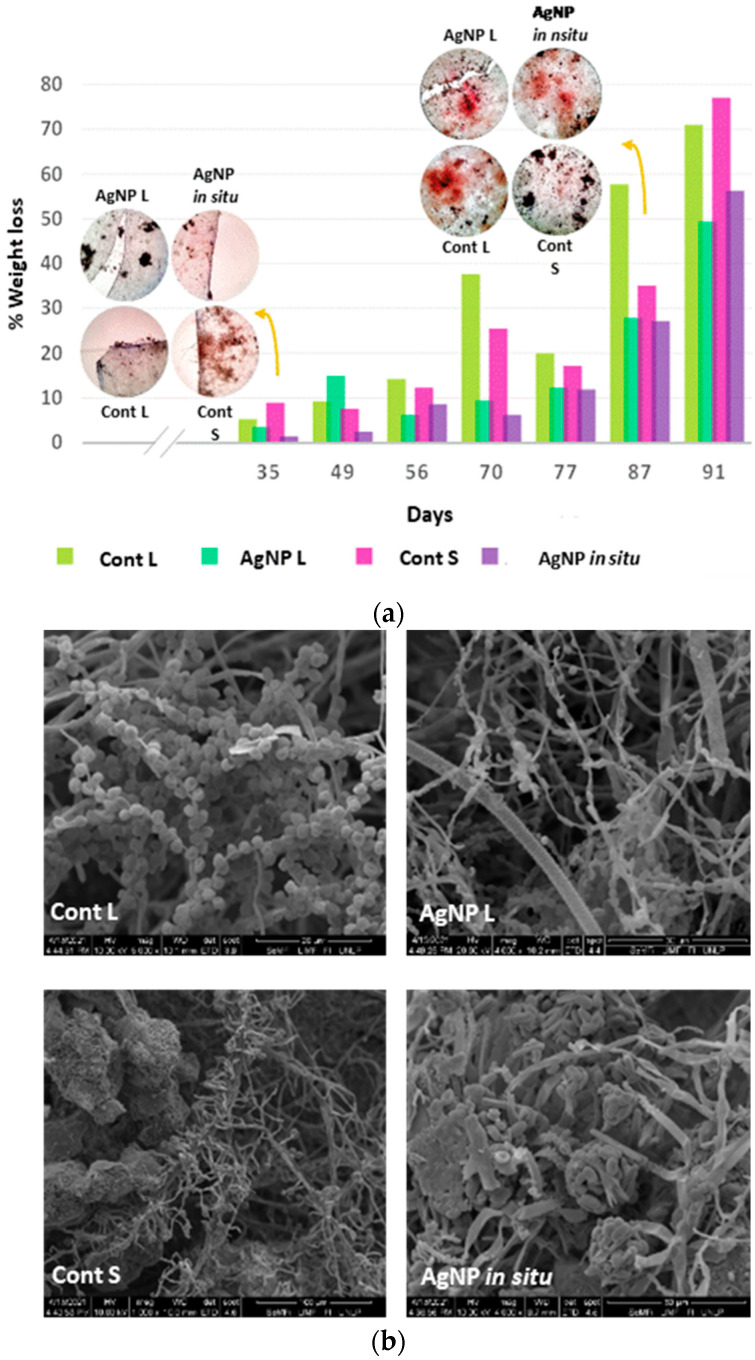
Weight loss percentage of the materials subjected to bio-disintegration: control and nanocomposite films with AgNP L and AgNP in situ (**a**). Control and nanocomposite film samples after 87 days of disintegration observed with the scanning electron microscope (**b**).

When analyzing the percentage of weight loss during the bio-disintegration assay, it was found that for control samples, this value is around 74% after 91 days of being buried in compost and, for the same period of time, this result was 49.4% for the samples with AgNP L and 56.3% for the films with nanoparticles synthesized in situ. The presence of hyphae or mycelial growth can be observed in the microscope images inserted in Figure 6a and in SEM micrographs (Figure 6b), which supports the aforementioned results and agrees with those observed in Figure 5. 

#### Compost Ecotoxicity Analysis

As mentioned by Balaguer et al. [53], not all biodegradable polymers are compostable, since compostability implies biodegradation by biological processes at a rate consistent with other known compostable materials, leaving non-visibly distinguishable or non-toxic residues. Thus, evaluation of compostability includes three phases: disintegration, biodegradation and ecotoxicity. The phytotoxicological aspect of the degradation process of biodegradable plastics is extremely important because plants are at the bottom of the food chain in the ecosystem [54]. According to this, ecotoxicity tests were performed in order to observe possible adverse effects resulting from the degradation of the films containing AgNPs in soil at the end of the useful life. Soil samples supplemented with the rest of the nanocomposite films were tested after biodegradation using the plant growth assay.

**Figure 7:**
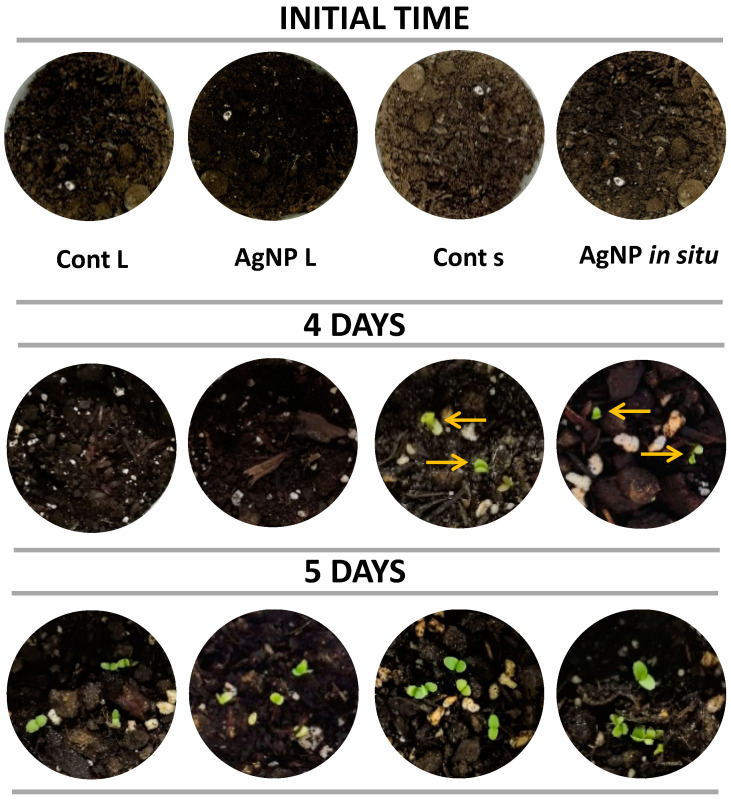
Evaluation the phytotoxic effect of the compost derived from the bio-disintegration of nanocomposite films and control films on the germination of lettuce seeds.

The germination of lettuce seeds, selected by their high growth rate, was carried out under environmental conditions and in all cases sprouts were observed. In the case of the compost derived from control materials and that in contact with films containing silver nanoparticles synthesized in situ, 4 days were required to detect the sprouts, while the rest of the samples germinated after 5 days (Figure 7). Thus, it can be inferred that the films’ biodegradation did not contribute compounds with phytotoxic activity to the compost under the evaluated conditions.

## 4. Conclusions

The migration of nanoparticles (AgNP in situ and AgNP L) from the nanocomposite material to three food simulants was studied, modeling the obtained results through different widely accepted models to describe this process. Regarding the content of Ag released to the simulant (mg of Ag that migrated per kg of simulant), it was higher for the films with AgNPs synthesized in situ than for those containing AgNP L. This was also associated with the different concentrations of Ag that both nanocomposite materials contain, as can be expected. Regardless of the maximum values obtained, these are below the limit established by current legislation. Although the Ag migration process was studied using food simulants, the results obtained overestimate the real application conditions in a foodstuff, and in this sense it would be encouraging regarding the materials’ safety.

The bio-disintegration of films in soil is a complex process that should be approached from several complementary analyses, such as the evaluation of the visual and structural appearance of the buried material, weight loss occurrence and microbial attack, as well as the final compost quality. The bio-disintegration of the developed materials was demonstrated in a period of 90 days. In addition, the ecotoxicity test allowed us to infer that the bio-disintegration of the studied films did not contribute substances with phytotoxic activity to the compost under the environmental conditions evaluated, allowing the germination of fast-growing species that are indicators of phytotoxicity.

Finally, the obtained results allow us to infer that both developed nanocomposite materials (AgNP in situ and AgNP L) would be useful as food packaging, considering the low migration potential in food simulants, which is within the permitted legislation limits and their high bio-disintegration rate. 

## Figures and Tables

**Table 1:** Thickness, water vapor permeability (WVP) and mechanical properties of nanocomposite starch films.

Starch Film Sample	Thickness (μm)	WVP (10^−10^ g/m s Pa)	Mechanical Properties		
			E(%)	TS (MPa)	EM (MPa)
AgNP in situ	97.3 ± 1.2 ^b^	1.38 ± 0.05 ^b^	32.5 ± 0.7 ^a^	5.8 ± 0.3 ^c^	15.2 ± 1.3 ^b^
AgNP L	102.7 ± 3.9 ^c^	0.63 ± 0.07 ^a^	40.0 ± 5.6 ^b^	4.0 ± 0.6 ^b^	14.2 ± 1.8 ^b^
Control	87.9 ± 5.0 ^a^	2.9 ± 0.1 ^c^	32.7 ± 0.8 ^a^	2.9 ± 0.2 ^a^	3.7 ± 0.6 ^a^

E: elongation at break, TS: tensile strength and EM: elasticity modulus). Means ± SD values are presented. Different letters within the same column indicate significant differences (*p* < 0.05).

**Table 2:** Kinetic model parameters of Ag release from nanocomposite starch films in selected food simulants.

Model	Water		3% *v*/*v* Acetic Acid		15% *v*/*v* Ethanol	
	AgNP L	AgNP In Situ	AgNP L	AgNP In Situ	AgNP L	AgNP In Situ
**Diffusion**						
D_1_ ^a^ (cm^2^/s)	5.61 × 10^−10^	3.41 × 10^−9^	1.53 × 10^−9^	1.99 × 10^−9^	3.72 × 10^−9^	3.68 × 10^−9^
*R* ^2 b^	0.91	0.98	0.92	0.98	0.98	0.98
D_2_^c^ (cm^2^/s)	1.33 × 10^−7^	1.84 × 10^−9^	1.02 × 10^−9^	1.14 × 10^−9^	1.77 × 10^−9^	3.57 × 10^−10^
*R* ^2 b^	0.96	0.73	0.65	0.52	0.82	0.74
**Ritger and Peppas**						
*n*	0.20	0.59	0.21	0.23	0.61	0.43
*k*	0.55	0.08	0.30	0.28	0.06	0.06
MSE ^d^	8.55 × 10^−6^	5.5 × 10^−4^	1.9 × 10^−3^	1.4 × 10^−3^	1.4 × 10^−3^	3.8 × 10^−5^
**Weibull**						
*a*	0.84	0.06	0.32	0.27	0.05	0.02
*b*	0.22	0.74	0.36	0.47	0.79	1.02
MSE ^d^	2.2 × 10^−3^	1.2 × 10^−3^	0.01	2.4 × 10^−3^	2.1 × 10^−3^	2.8 × 10^−3^

^a^ D_1_: corresponds to the diffusion coefficient of the data fitted with Equation (2). ^b^ Correlation coefficient. ^c^ D_2_: corresponds to the diffusion coefficient of the data fitted with Equation (3) at short contact times. ^d^ Mean square error. The smaller the MSE values, the greater the goodness of fit of the experimental data to the proposed model [43].

## Data Availability

Data will be available on demand.
